# Testing the feasibility of blood flow restriction training to enhance the HEALTH benefits of exercise in individuals with type 2 diabetes (BOOST-HEALTH Trial): Study Protocol

**DOI:** 10.1371/journal.pone.0346176

**Published:** 2026-03-30

**Authors:** Amy M Thomson, Seth F McCarthy, Jamie F Burr, Jonathan P Little, Maryam Kebbe, Martin Sénéchal

**Affiliations:** 1 Cardiometabolic Exercise & Lifestyle Laboratory, Fredericton, New Brunswick, Canada; 2 Faculty of Kinesiology, University of New Brunswick, Fredericton, New Brunswick, Canada; 3 Exercise, Metabolism and Inflammation Lab, Kelowna, British Columbia, Canada; 4 School of Health and Exercise Sciences, University of British Columbia, Kelowna, British Columbia, Canada; 5 Human Performance & Health Research Laboratory, Guelph, Ontario, Canada; 6 Human Health and Nutritional Sciences, University of Guelph, Guelph, Ontario, Canada; PLOS: Public Library of Science, UNITED KINGDOM OF GREAT BRITAIN AND NORTHERN IRELAND

## Abstract

**Introduction:**

Individuals with type 2 diabetes (T2D) display reduced cardiorespiratory fitness, a strong predictor of premature mortality and T2D-related complications. Aerobic training (AT) enhances cardiorespiratory fitness and is a cornerstone in T2D management. Emerging data suggest that AT combined with blood flow restriction (AT+BFR) may elicit greater improvements in cardiorespiratory fitness than typical AT in healthy individuals. However, the feasibility and effects of AT+BFR in individuals with T2D remain unclear.

**Objectives:**

This protocol describes the BOOST-HEALTH trial, which aims to 1) evaluate the feasibility of a 6-week AT+BFR intervention in adults with T2D, and 2) estimate preliminary effect sizes for changes in cardiorespiratory fitness, glycemic outcomes, and quality of life compared with standard care AT (AT- stdCare).

**Methods:**

BOOST-HEALTH (NCT07196371) is a single-blinded, multisite, randomized clinical pilot trial with two parallel treatment arms. Sixty adults with T2D (n = 30 female) will be randomized to 6 weeks of AT+BFR or AT-stdCare. Both groups will complete supervised treadmill walking three times per week (96 min/week) at 40–50% heart rate reserve. The AT+BFR group will exercise with BFR cuffs inflated to 60–80% of limb arterial occlusion pressure, whereas the AT-stdCare group will exercise without BFR. Primary outcomes will assess feasibility, including recruitment, enrollment, adherence, and retention. Secondary outcomes will estimate effect sizes for changes in cardiorespiratory fitness (VO_2max_) and continuous glucose monitoring metrics to inform outcome selection and sample size calculations for future definitive trials. Outcomes will be assessed at baseline and post-intervention.

**Discussion:**

Results of this study could establish BFR training as a novel therapeutic modality to augment the effects of exercise in individuals with T2D. Findings will inform whether a larger, definitive efficacy trial is warranted and will guide exercise professionals on the potential integration of AT+BFR as an alternative strategy to enhance the benefits of exercise in this population.

## Introduction

The global burden of diabetes continues to grow at an alarming rate, with over 500 million people affected worldwide and projections indicating a 150% increase by 2050 [1]. In Canada, there are approximately 4 million individuals diagnosed with diabetes, with type 2 diabetes (T2D) accounting for over 90% of all cases [2,3]. Diabetes is the seventh leading cause of death in Canada [4], reduces lifespan by 5–15 years [4,5], and incurs lifetime direct treatment costs exceeding $125,000 in adults, placing immense strain on both the healthcare system and population health [6]. In light of its impact on morbidity, mortality, and healthcare resource utilization, there is an urgent need for novel strategies that optimize T2D management and reduce long-term expenditures.

Exercise remains a cornerstone in managing T2D, with broad consensus across international organizations recognizing its importance [7,8] and substantial evidence supporting its role in improving glycemia, insulin sensitivity, cardiorespiratory fitness, and overall metabolic health [9–19]. Lifestyle interventions for T2D that involve regular physical activity are associated with a 10–25% reduction in all-cause mortality [20–25], with many of these benefits from aerobic exercise attributed to improvements in cardiorespiratory fitness [26–30]. Despite the recognized benefits of exercise in T2D, responses to exercise interventions are not uniform across all individuals, with substantial inter-individual variability in metabolic and physiological responsiveness to exercise [31–33]. Compounding these challenges, individuals living with T2D exhibit an approximately 30% reduction in exercise tolerance compared to counterparts without diabetes [34], suggesting that simply increasing exercise intensity or volume may not be an appropriate route to improve cardiorespiratory fitness. These data underscore the need for alternative exercise strategies that can overcome this blunted physiological response.

To address this challenge, novel approaches that enhance the physiological stimulus of exercise without increasing the overall volume or intensity are urgently needed. Blood flow restriction (BFR) training has emerged as an innovative modality of exercise that may present a promising strategy to optimize exercise prescriptions for T2D management [35]. BFR involves the application of controlled pressure to the limbs during exercise using a tourniquet system to partially restrict arterial inflow and occlude venous return [36], which amplifies metabolic stress and local hypoxia, contributing to physiological adaptations. While originally used in healthy and athletic populations [37,38], emerging evidence supports BFR’s safety and efficacy in clinical populations with neurological and musculoskeletal disorders [39]. When combined with resistance or aerobic exercise, BFR enhances muscular and cardiovascular adaptations at significantly lower intensities than traditional training [36,40–45]. Similarly, low-to-moderate intensity aerobic exercise performed with BFR can augment improvements in cardiorespiratory fitness, strength, and lean mass compared to aerobic exercise alone [46–49]. Altogether, in the context of T2D, BFR is an exciting avenue as it has the capacity to increase skeletal muscle mass, the main site of glucose uptake [50], and increase cardiorespiratory fitness [46,48,49]. However, to the best of our knowledge, no trials have examined the feasibility or efficacy of treadmill-based aerobic training + BFR training in individuals of both sexes living with T2D [35, 51–54].

Given the impaired cardiorespiratory fitness, substantial heterogeneity in exercise responses, and elevated cardiometabolic risk observed in this population, BFR may offer a novel and practical method to amplify exercise adaptations without exceeding tolerable workloads. Therefore, the primary objective of this pilot trial is to assess the feasibility of a six-week moderate-intensity aerobic training (AT)+BFR intervention in individuals living with T2D. The secondary objective is to establish effect sizes for the efficacy of AT+BFR compared with aerobic training standard care (AT-stdCare) for changes in cardiorespiratory fitness, glycemia, and quality of life measures in individuals living with T2D. We hypothesize that AT+BFR training will elicit superior improvements in cardiorespiratory fitness compared with traditional aerobic exercise, thereby offering a novel approach to enhance the impact of lifestyle interventions in T2D care.

## Methods and analysis

### Trial design

The BOOST-HEALTH trial is a single-blinded, multisite, randomized clinical pilot trial with two parallel treatment arms examining the feasibility of aerobic training with BFR in adults living with T2D. Eligible participants will be randomized into one of two intervention arms: 1) aerobic training + blood flow restriction (AT+BFR), or 2) aerobic training + standard care (AT-stdCare).

### Trial setting

The BOOST-HEALTH trial is a Canadian study that will be conducted in the Cardiometabolic Exercise and Lifestyle Laboratory (CELLAB) in the Faculty of Kinesiology at the University of New Brunswick, the Exercise, Metabolism and Inflammation Lab (EMIL) in the Faculty of Health and Social Development at the University of British Columbia Okanagan, and the Human Performance and Health Research Lab (HPHL) in the Department of Human Health Sciences at the University of Guelph, Ontario. These locations (Fredericton, New Brunswick; Okanagan, British Columbia; Guelph, Ontario) were selected based on the availability of dedicated exercise testing and training facilities, appropriate equipment, capacity to support larger-scale trials, a strong history of cardiometabolic research in exercise, and access to research personnel with expertise in clinical trial implementation and experience working with individuals living with T2D.

### Eligibility criteria

#### Inclusion criteria.

1. Community-dwelling adults aged 19–64 years.
2. Previously diagnosed with T2D and current glycated hemoglobin (HbA1c) value of between 5.7-9.0%.
3. Not currently partaking in regular physical activity, defined as engaging in <150 minutes of moderate-to-vigorous aerobic activity per week, confirmed via the Godin Leisure-Time Exercise Questionnaire and the Get Active Questionnaire.

#### Exclusion criteria.

1. A self-reported diagnosis of low iron concentrations, anemia, or being treated for these conditions.
2. A diagnosis of any red blood cell-altering condition (i.e., sickle cell anemia, poikilocytosis).
3. Currently prescribed any medication that would impact the ability to use a heart rate monitor to accurately track exercise (i.e., beta-blockers).
4. Unstable T2D medications over the past 3 months.
5. Any musculoskeletal issues or injuries preventing exercise training.
6. Any absolute contraindications to BFR (i.e., peripheral vascular disease, history of MI) [55,56].
7. Any relative contraindications to BFR and deemed unsafe to participate when reviewed by the study clinician [55,56].

### Recruitment

Participants (n = 60; n = 20 per site) will be recruited via social media and radio advertisements, as well as by advertisements placed in pharmacies, healthcare centers, physician offices, and community organizations. Further recruitment will occur through electronic communication, including e-newsletters within various organizations and groups. Participants from previous studies who expressed interest in being considered for future research will also be contacted.

The BOOST-HEALTH trial began participant recruitment on January 1^st,^ 2026. The anticipated completion date of all data collection and follow-up visits is December 31^st^, 2027. Results from the trial are expected to be available by December 2028.

### Intervention

The intervention will involve 6 weeks of treadmill-based aerobic exercise at 40–50% of heart rate reserve (HRR). The AT+BFR participants will perform the treadmill-based aerobic exercise with BFR cuffs inflated around the proximal thigh at 60–80% of their individualized limb occlusion pressure (LOP) determined at rest before each session using an automated BFR tourniquet system (PTSi, Delfi Medical Innovations Inc. Vancouver, Canada). The percentage of LOP will progressively increase over the course of the intervention. Weeks 1 and 2 will be completed at 60% LOP, after which pressure will increase by 5% each subsequent week, corresponding to 65%, 70%, 75%, and 80% LOP during weeks 3, 4, 5, and 6, respectively. The BFR cuffs will remain inflated throughout the exercise protocol, but will deflate for 1 minute every 10 minutes as per safety/tolerability recommendations [55]. The AT-stdCare participants will follow the same training schedule and progression as the AT+BFR group without using BFR cuffs during the exercise sessions.

Participants will be eased into the program using a 1-week progressive start; they will complete 63 minutes of exercise in Week 1, split across 3 sessions (21 mins/session). For the remaining 5 weeks, participants will complete 96 minutes of exercise split across 3 sessions per week (32 mins/session). Each exercise session will begin with a five-minute warmup to achieve target intensity and end with a five-minute cool down, neither of which will be counted in the total exercise time. All exercise sessions will be supervised by research staff and take place in a private exercise facility located at each site. To maximize adherence to the intervention, exercise sessions are scheduled on a weekly basis with research staff available 7 days a week.

### Exercise monitoring

To ensure participants are exercising at the appropriate intensity, research staff will set the treadmill at the speed and grade designed to elicit their 40–50% HRR without BFR. Research staff will supervise each session and record participant heart rate every 5 minutes using the Polar Team2 (Polar, Kempele, Finland) system, along with treadmill speed and slope. The supervising staff will ensure the participants maintain their prescribed intensity and that the BFR cuffs are deflated and re-inflated as per the protocol. Additionally, participants’ resting blood pressure will be recorded before and after each exercise session.

### Deviations from protocol

Research staff will ensure that each participant receives the same dose of exercise. If a participant misses their training sessions for an entire week (due to illness, family emergency, etc.), up to two additional weeks may be added to the end of the intervention for each missed week. The reason provided for missing a week of training will be documented and available for interpretation and analysis purposes. If a participant is unable to complete the required three sessions of aerobic training after starting a given week of exercise (due to illness, injury, emergency, etc.), any remaining session(s) will be carried forward and completed during the subsequent week of the intervention. From the date of the first exercise session, participants will have a maximum of 8 weeks to complete all 18 prescribed exercise sessions. If a participant exceeds the 8-week period without completing all 18 sessions, the total number of sessions completed will be recorded. Enrollment in the trial will be discontinued if a participant experiences an injury or medical event that would limit safe participation or require medical attention, or if the participant receives medical advice to withdraw from the trial. These types of events will be closely monitored and documented in accordance with the feasibility outcomes.

### Data collection and management

**Fig 1:**
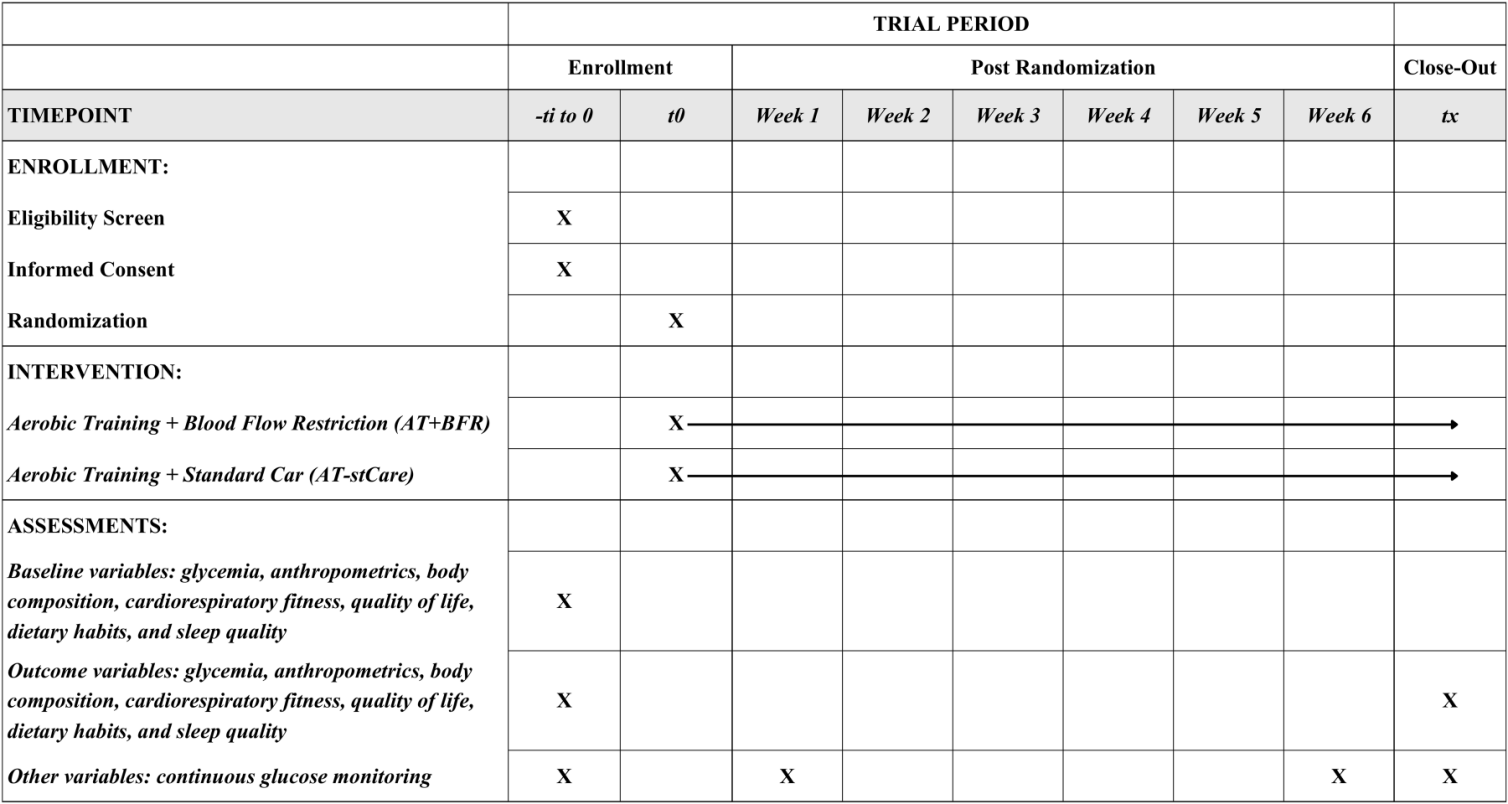
SPIRIT Participant Timeline: Schedule of enrollment, interventions, and assessments. -t_i to 0_: time before randomization consisting of a phone screening for eligibility and two baseline testing visits, t_0_: time of randomization, t_x_: post-testing consisting of two visits after the intervention.

At the time of first contact with research staff, participants will be assigned a unique identifier (ID), and all files will subsequently be deidentified. Participants will meet with research staff for the purpose of data collection, outside of exercise sessions, a total of four times: twice at baseline testing and twice at post-testing (Fig 1). All data obtained from baseline and post-intervention testing visits will be collected in written form and then transferred to electronic files. All other data collected throughout the intervention will be collected electronically. Physical versions of files will be stored locally in a locked cabinet in a locked room in a restricted access research lab at each site, while digital files will be password-protected and secured on Research Electronic Data Capture (REDCap) tools hosted at the University of British Columbia (Research Electronic Data Capture, Nashville, USA) [57,58].

### Outcomes and instrumentation

#### Primary outcome.

The primary outcome of the BOOST-HEALTH Trial is the feasibility of a larger definitive trial. Primary selected end-points used to determine feasibility will include: 1) recruitment rates defined as the number of individuals inquiring about the trial, 2) enrollment rates defined as the number of participants who consent to participate in the trial and are randomized to one of the two study arms, 3) adherence to the intervention arms defined as the number of sessions each participant attended during the trial, 4) retention for follow-up testing defined as the number of participants who complete all post-testing measurements after the intervention, and 5) adverse events defined as any undesirable event occurring during the study (e.g., pain, fatigue, fracture) [59]. We will aim to recruit at least 1 participant per month per site, achieve >70% adherence, retain >80% of participants for follow-up testing, and minimize any adverse events related to BFR or AT.

#### Secondary outcomes.

Cardiorespiratory fitness (VO_2peak_) will be assessed using a modified Balke and Ware treadmill test protocol. Participants will walk at self-selected speed between 4.5-5.5 kilometers per hour (km/h) at a 0% grade on a motorized treadmill. After 2 minutes, the grade will be increased to 5.0% for 2 minutes, and then progressively increase by 1% every minute until a maximum grade of 15% is achieved. If a participant reaches the maximum grade, then speed will increase by 0.8 km/h each minute until volitational fatigue. Gas exchange will be continuously monitored using a metabolic cart, heart rate data will be obtained throughout the test using a Polar FT1 heart rate monitor (Polar, Kempele, Finland), and blood pressure data will be recorded every 2 minutes using a digital blood pressure monitor. VO_2peak_ will be identified as the average of the highest 30 seconds of VO_2_ observed during the last minute of the test.

Glycemia will be assessed through glycated hemoglobin (HbA1c) using the Abbott Afinion^TM 2^ system analyzer. A 1-µL capillary whole blood sample will be obtained via finger prick using a single-use lancet and undergo rapid assessment of HbA1c in the point-of-care analyzer, with results available in approximately 3 minutes. In addition, blood glucose will be measured using commercially available continuous glucose monitors (CGMs) (Abbott, FreeStyle Libre Pro 3) for 15 days (7 days prior to the intervention and the first 8 days of the intervention/ last 8 days of the intervention and 7 days post intervention). CGM data will be analyzed using the Diametrics platform [60] yielding outcomes as described in the consensus for reporting of CGM data in trials [61].

Quality of Life will be assessed using validated self-report questionnaires. Health-related quality of life will be measured using the 36-Item Short-Form Health Survey (SF-36), which comprises eight domains: physical functioning, role limitations due to physical health, role limitations due to emotional problems, vitality, emotional well-being, social functioning, pain, and general health perceptions. Each domain is scored from 0 to 100, with higher scores indicating better quality of life [62]. Diabetes-specific impacts on quality of life will be assessed using select questionnaires from the American Diabetes Association Behavioural Health Toolkit [63]. Diabetes-related distress will be measured using the Problem Areas in Diabetes (PAID) Scale [64], a 20-item questionnaire, and the Diabetes Distress Scale (DDS-17) [65], which includes 17 items across 4 subscales: emotional burden, regimen-related distress, interpersonal distress, and physical distress. Higher scores on both instruments indicate greater diabetes-related distress. Fear of hypoglycemia will be assessed using the Hypoglycemia Fear Survey-II (HFS-II W) [66], a 33-item questionnaire consisting of behaviour worry subscales, with higher scores reflecting greater concern related to hypoglycemia. Perceptions of insulin therapy will be evaluated using the Insulin Treatment Appraisal Scale (ITAS) [67], which includes 20 items comprising four positive and 16 negative appraisal statements; higher total scores indicate more negative attitudes toward insulin use. Symptoms of depression and anxiety will be assessed using the Patient Health Questionnaire Nine (PHQ-9) [68] and the Generalized Anxiety Disorder Seven (GAD-7) [69], respectively. The PHQ-9 consists of nine items assessing depressive symptoms and their severity, with scores ≥10 indicating likely depression. The GAD-7 includes seven items assessing anxiety symptoms and their severity, with scores ≥10 indicating a likely anxiety disorder.

#### Exploratory outcomes.

At baseline, research staff will record participant self-reported demographics (age, sex, gender, ethnicity, socioeconomic status, and education), family medical history, T2D duration, and current medication usage. Participants will be monitored throughout the study and asked to report any changes in medication use to research staff as they occur. Medication usage and changes will be confirmed through detailed label inspection (of bottle or photo) or a pharmacy printout.

Physiological and anthropometric measurements will be taken over the span of two days, separated by less than one week. Participants’ height, weight, resting blood pressure, resting heart rate, and hip and waist circumference will be measured by a member of the research staff according to the Canadian Society for Exercise Physiology protocols [70]. Briefly, participants’ height and weight will be measured to the nearest 0.5 cm and 0.1 kg using a stadiometer and calibrated column scale, respectively. For the height measurement, participants will be asked to stand straight with their feet together, arms at their sides, with no shoes, and the measurement will be taken following an inhalation [71]. BMI will be calculated from the height and weight measurements (BMI = kg/m^2^). Waist circumference will be measured at the upper lateral border of the iliac crest at the end of a normal expiration while the participant stands with their feet shoulder-width apart. The average of two measurements, to the nearest 0.5 cm, will be recorded with an anthropometric tape measure [71]. If the two measures differ by greater than 1 cm, a third measure will be performed, and the average of the two closest measures will be recorded. Hip circumference will be measured around the maximal circumference of the buttocks, following the same protocol as waist circumference [72]. Resting blood pressure and heart rate will be measured twice while the participant is seated, following at least five minutes of rest, using a digital blood pressure monitor [71]. The average of the two measures will be recorded.

Body composition, including fat mass, lean mass, and body fat percentage, will be estimated using dual-energy x-ray absorptiometry (DXA) following a 12-hour overnight fast. Participants will be instructed to present to the CELLAB, EMIL, or HPHL wearing loose-fitting clothing with no metal (buckles, zippers, etc.) and to then lie supine on the DXA table and remain still for the duration of the scan. Arms will be placed at the participants’ sides with palms facing medially and thumbs pointed upwards.

Blood samples will be collected intravenously from the antecubital vein by a registered nurse or certified phlebotomist into 3mL Vacutainer collection tubes coated with an anticoagulant, ethylenediaminetetraacetic acid (EDTA). The blood samples will then be centrifuged at 1600g for 15 minutes (4°C) and the plasma aliquoted into 1.5mL microcentrifuge tubes for storage at −80°C until further analysis.

Dietary information will be recorded using the Automated Self-Administered 24-Hour Dietary Assessment Tool (ASA24), reporting a weekday and weekend day at both baseline and post-testing to account for potential dietary intake variability [73]. However, participants will be instructed to not change their dietary habits during the study. The ASA24 will be used to quantify total energy intake and macronutrient distribution. The instrument has been found to be a valid measure as well as reliable, balancing participant burden and time [73–75].

Sleep quality will be measured at each timepoint using the Pittsburgh Sleep Quality Index (PSQI) [76]. The PSQI consists of 19 items encompassing seven subcategories: subjective sleep quality, sleep latency, sleep duration, habitual sleep efficiency, sleep disturbances, use of sleeping medication, and daytime dysfunction, which are summed into a global sleep score. The global sleep score ranges from 0-21, with scores ≥5 indicative of poor sleep quality [76].

### Blinding

To maintain single blinding, principal and co-investigators and the statistician will be blinded to participant study groups for data analyses. It is not feasible to blind participants in this trial.

### Randomization

Randomization of the intervention participants will occur following completion of the baseline testing visits. Participants will be randomized using a 1:1 allocation ratio with variable permuted block sizes (stratified by sex and site) through the web-based platform REDCap. When a new participant completes baseline testing and is ready for randomization, the participant’s ID number, sex, and location will be entered into REDCap, which will display the randomization (AT+BFR or AT-stdCare) for that participant.

### Sample size calculation

There are no clear guidelines for calculating sample size for a pilot/feasibility trial. The sample size calculation was based on determining the feasibility of a larger trial and informed by control group and BFR group data from a study performed by one of our team members using cardiorespiratory fitness as a key efficacy outcome [46]. The mean change in cardiorespiratory fitness for the control and the BFR training group were 1.3% + 4.8% and 8.7% + 7.3%, respectively, from which an effect size Cohen’s d was calculated to be 1.2. To be more conservative, using an effect size of 0.25 and nominal type 1 (0.05) and type 2 (0.10) errors, our sample size was estimated to be a total of 46 (n = 23 per arm) for a repeated-measures, within-between interaction (calculated using G*Power v3.1). To account for a potential 25% dropout rate, which is typical for exercise trials [77], and missing data, a total sample size of n = 60 (n = 30 per arm; n = 20 per site) was selected.

### Statistical analysis

The normality of the data will be assessed using the Kolmogorov-Smirnov test and confirmed with a visual inspection of the data. General characteristics of the sample will be presented as mean ± standard deviation (SD) for continuous variables and n (%) for categorical variables. As per the CONSORT statement guidelines, changes in outcomes for this trial will be reported as mean changes and 95% confidence intervals (95% CI). Primary outcome analyses will involve an intention-to-treat strategy with no imputation of missing data. Secondary outcomes (VO_2peak_, CGM, QoL) will be tested for group-wise differences using a mixed effects regression model with a random effect for participants to account for the repeated measures and fixed effects for group, time, and their interaction. The main effect estimate of interest from the model is the between-group difference at follow-up. Stratification variables (sex, site) and covariates (age, baseline values) will be included as appropriate, depending on the distribution of the data and relationship to individual outcomes. All data management will be performed using the REDCap platform to compile the data from all three sites. Statistical analyses will be performed using SPSS version 16 and STATA/SE version 16.1. A *p*-value of less than or equal to 0.05 (*p* ≤ 0.05) will be considered significant.

### Data monitoring

A data monitoring committee was not included as this trial involves a behavioural intervention with known/minimal risks and does not require periodic benefit-risk assessments. The principal investigators from each site will meet monthly with the study physician and select research staff to discuss trial progression. The level of risk for adverse events associated with participation in this trial is considered low. If an adverse event occurs, it will be recorded and reported to the site principal investigator, who will inform the other site principal investigators and the institutional Research Ethics Board. No independent auditing of trial conduct is planned.

### Involvement of persons with lived experience

Persons with lived experience (PWLE) of T2D were actively engaged in the development of this trial. Using the *Theoretical Framework of Acceptability*, we consulted older adults living with T2D who had previously completed six weeks of BFR resistance training [78]. Their lived experience informed key design decisions, including intervention feasibility and perceived benefits, and directly shaped the trial methodology. PWLE feedback indicated that BFR training was acceptable, comfortable after initial exposure, and associated with rapid improvements in perceived physical function. PWLE will continue to be meaningfully engaged throughout the study as members of a patient advisory committee. This committee will meet throughout the trial to advise on recruitment, adherence, and knowledge mobilization. PWLE will also contribute to dissemination activities and knowledge translation efforts.

### Ethics

All experimental procedures have been approved by the Research Ethics Board at the University of New Brunswick (REB 2025–069), the University of British Columbia (REB: H25-02870) and the University of Guelph (REB #2035). Any substantial protocol amendments will be submitted to each Research Ethics Board for review and approval prior to implementation and will be clearly documented in a protocol deviation document.

### Informed consent

Upon initial contact with research staff, interested individuals will be provided with key information pertaining to the BOOST-HEALTH trial and have their eligibility confirmed through a phone screening. Prior to the first baseline testing visit, where eligibility will be confirmed, participants will be provided with a digital copy of the consent form to review. At the beginning of the first baseline testing visit, eligible participants will have time to review a physical copy of the consent form, ask any questions, and consider their participation. If the participant decides to proceed with participating in the study, they will be asked to provide written informed consent, which will be cosigned by research staff. All participants are free to withdraw from the study at any time.

### Dissemination

Results from the BOOST-HEALTH trial will be submitted to peer-reviewed journals and presented at scientific meetings. The findings from this study will be used to support and drive future randomized trials exploring the efficacy of BFR training for individuals living with T2D. Participants will have the opportunity at the time of consent to request a copy of the study findings. Upon completion of the trial, a summary of findings and personalized report will be provided to those participants. Members of a patient advisory committee will inform the research team on the potential best ways to disseminate the results of this study to the patients and the general public.

### Trial status

The BOOST-HEALTH trial was registered at ClinicalTrials.gov (NCT07196371) in September 2025.

## Supporting information

S1 Checklist SPIRIT 2025 checklist. (DOCX)

S1 File Clinical trial protocol. (PDF)
