# Supplementary material for: Testing the feasibility of blood flow restriction training to enhance the HEALTH benefits of exercise in individuals with type 2 diabetes (BOOST-HEALTH Trial): Study Protocol
Source: PLoS One. 2026 Mar 30;21(3):e0346176. doi: 10.1371/journal.pone.0346176 (PMC13035122; doi:10.1371/journal.pone.0346176)
Supplement: S1 File — (PDF) [file pone.0346176.s002.pdf]

Research Protocol

Overview

Rationale: Individuals with type 2 diabetes (T2D) display reduced cardiorespiratory fitness, which is a strong predictor of premature mortality and T2D-related complications. Aerobic training (AT) enhances cardiorespiratory fitness and is a cornerstone in the management and treatment of T2D. Emerging data suggest that AT combined with blood flow restriction (AT+BFR) training could enhance cardiorespiratory fitness faster than typical AT in healthy individuals. However, the feasibility of AT+BFR in individuals with T2D and its impact on cardiorespiratory fitness have yet to be determined.

Objectives: The primary objective of this study is to test the feasibility of a 6-week AT+BFR training intervention in individuals with T2D. The secondary objective is to establish preliminary effect sizes for the efficacy of AT+BFR training in individuals with T2D compared to standard care AT (AT- stdCare).

Study Design: This study is a single-blind (investigator & statistical analyst), multi-site, randomized controlled pilot trial of a novel AT+BFR training intervention for individuals living with T2D, informed by patients with lived experience. Participants will be randomized 1:1 using variable permuted block sizes (stratified by sex and site) into AT+BFR or AT-stdCare groups with outcome measures assessed at baseline and 6 weeks.

Intervention: The intervention will consist of 6 weeks of AT+BFR. Participants will perform AT+BFR 3 times weekly for 21-32 minutes per session. A blood pressure cuff will be set at 60-80% of limb arterial occlusion with AT progressing to 96 minutes per week of treadmill walking performed at 40-50% of heart rate reserve (HRR).

Control: Participants in the AT-stdCare will perform 96 minutes per week of treadmill walking at 40-50% of HRR following the same schedule as AT+BFR, training 3 times per week for 21-32 minutes per session.

Outcomes: The main outcome measures will pertain to the feasibility of a larger trial and include recruitment rates, enrollment and adherence to the intervention, and retention for follow-up testing. The secondary outcomes will be focused on establishing effect sizes to power a larger trial. Effect sizes for changes in cardiorespiratory fitness and continuous glucose monitoring (CGM) outcomes will be determined to help select and power a primary outcome for a more definitive trial of AT+BFR. Exploratory outcomes will include quality of life measured with the American Diabetes Association Mental Health Tool Kit, sleep quality using the Pittsburgh sleep questionnaires, and dietary habits using the ASA24 self-administered 24-hour diet recall.

Impact: This study will be the first feasibility trial to test blood flow restriction as a strategy to augment the effects of exercise in individuals with T2D and could establish BFR training as a novel therapeutic modality for this population. Findings will determine if a larger, more definitive efficacy trial is warranted and will inform exercise specialists of an innovative exercise alternative to maximize the benefits of exercise in individuals living with T2D.

Background: The global burden of diabetes continues to grow at an alarming rate, with over 500 million people affected worldwide and projections indicating a 150% increase by 2050[1]. In Canada, approximately 6 million individuals live with diabetes, with type 2 diabetes (T2D) accounting for over 90% of all cases[2]. Diabetes is the seventh leading cause of death in Canada[3], reduces lifespan by 5 to 15 years[3,4] and incurs lifetime direct treatment costs exceeding $125,000 in adults, placing immense strain on both the healthcare system and population health[5]. In light of its impact on morbidity, mortality, and healthcare resources, there is an urgent need for novel strategies that optimize T2D management and reduce long-term expenditures.

Exercise remains a cornerstone in managing T2D, with broad consensus across international organizations recognizing its importance[6,7] and substantial evidence supporting its role in improving glycemia, insulin sensitivity, cardiorespiratory fitness, and overall metabolic health[8–18]. Lifestyle interventions for T2D that involve regular physical activity are associated with a 10-25% reduction in all-cause mortality[19–24] with many of these benefits from aerobic exercise attributed to improvements in cardiorespiratory fitness[25–29].

Despite the recognized benefits of exercise in T2D, responses to exercise interventions are not uniform across all individuals, with substantial inter-individual variability in metabolic and physiological responsiveness to exercise[30–32]. Compounding these challenges, individuals living with T2D exhibit an approximately 30% reduction in exercise tolerance compared to counterparts without diabetes[33], suggesting that simply increasing exercise intensity or volume may not be an appropriate route to improve cardiorespiratory fitness. These data underscore the need for exercise strategies that can overcome this blunted physiological response.

To address this challenge, novel approaches that enhance the physiological stimulus of exercise without increasing the overall volume or intensity are urgently needed. Blood flow restriction (BFR) training has emerged as an innovative modality of exercise that may present a promising strategy to optimize exercise prescriptions for T2D management[34]. BFR involves the application of controlled pressure to the limbs during exercise using a tourniquet system to partially restrict arterial inflow and occlude venous return[35], which amplifies metabolic stress and local hypoxia, contributing to physiological adaptations. While originally used in healthy and athletic populations[36,37], emerging evidence supports its safety and efficacy in clinical populations with neurological and musculoskeletal disorders[38]. When combined with resistance or aerobic exercise, BFR enhances both muscular and cardiovascular adaptations at significantly lower intensities than traditional training[35,39–44]. Similarly, aerobic activity performed at low-to-moderate intensity combined with BFR has been shown to augment improvements in cardiorespiratory fitness compared to aerobic exercise alone[45–48], with some data suggesting an 8% increase in cardiorespiratory fitness after only two weeks of aerobic BFR training[45]. Altogether, in the context of T2D, BFR is an exciting avenue as it has the capacity to increase skeletal muscle mass, the main site of glucose uptake[49], and increase cardiorespiratory fitness[45,47,48]. However, to the best of our knowledge, no trials have examined the feasibility or efficacy of treadmill-based aerobic training + BFR training in individuals of both sexes living with T2D[34,50–53].

Given the impaired cardiorespiratory fitness, blunted responsiveness to standard aerobic training, and elevated cardiometabolic risk in this population, BFR may offer a novel and practical method to amplify exercise adaptations without exceeding tolerable workloads.

Objective: The primary objective of this pilot trial is to test the feasibility of a six-week moderate-intensity aerobic training (AT)+BFR intervention in individuals living with T2D. The secondary objective is to establish effect sizes for the efficacy of AT+BFR compared to AT standard care (AT-stdCare) for changes in cardiorespiratory fitness, glycemia, and quality of life measures in individuals living with T2D.

Hypothesis: We hypothesize that BFR training will elicit superior improvements in cardiorespiratory fitness compared to traditional aerobic exercise, thereby offering a novel and scalable approach to enhance the impact of lifestyle interventions in T2D care.

Methodology

Trial Design: The BOOST-HEALTH trial is a single-blinded, multisite, randomized clinical pilot trial with two parallel treatment arms examining the feasibility of aerobic training with BFR in adults living with T2D. Eligible participants will be randomized into one of two intervention arms: 1) aerobic training + blood flow restriction (AT+BFR), or 2) aerobic training + standard care (AT-stdCare).

Trial Setting: The BOOST-HEALTH Trial will be conducted in the Cardiometabolic Exercise and Lifestyle Laboratory (CELLAB) in the Faculty of Kinesiology at the University of New Brunswick, the Exercise, Metabolism and Inflammation Lab (EMIL) in the Faculty of Health and Social Development at the University of British Columbia Okanagan, and the Human Performance and Health Research Lab (HPHL) in the Department of Human Health Sciences at the University of Guelph, Ontario. These locations (Fredericton, New Brunswick; Okanagan, British Columbia; Guelph, Ontario) were selected based on the availability of dedicated exercise testing and training facilities, appropriate equipment, capacity to support larger-scale trials, a strong history of cardiometabolic research in exercise, and access to research personnel with expertise in clinical trial implementation and experience working with individuals living with T2D.

Inclusion Criteria: Participants will be eligible for inclusion if they are: 1) Community-dwelling adults aged 19-64 years, 2) currently living with T2D with a glycated hemoglobin (HbA1c) value between 5.7-9.0%, and 3) not currently partaking in regular physical activity.

Exclusion Criteria: Participants will not be eligible to participate if they are: 1) diagnosed with low iron concentrations, anemia, or are being treated for these conditions, 2) diagnosed with any red blood cell-altering condition (i.e., sickle cell anemia, poikilocytosis), 3) currently prescribed any medication that would impact the ability to use a heart rate monitor to accurately track exercise (i.e., beta-blockers), 4) diagnosed with any musculoskeletal issues or injuries preventing exercise training, 5) diagnosed with any absolute contraindications to BFR[54,55], 6) diagnosed with any

relative contraindications to BFR and deemed unsafe to participant when reviewed by a clinician[54,55], or 7) have unstable T2D medications over the past 3 months.

Recruitment: Participants (n = 60; n = 20 per site) will be recruited using social media and radio advertisements, and advertisements placed in pharmacies, healthcare centers, physician offices, and community organizations. Further recruitment will occur through electronic communication, including e-newsletters within various organizations and groups. Participants from previous studies who expressed interest in being considered for future research will also be contacted.

Intervention: The intervention will involve 6 weeks of treadmill-based aerobic exercise at 40-50% of heart rate reserve (HRR). The AT+BFR participants will perform the treadmill-based aerobic exercise with BFR cuffs inflated around the proximal thigh at 60-80% of their individualized limb occlusion pressure (LOP) determined at rest using an automated BFR tourniquet system (PTSi, Delfi Medical Innovations Inc. Vancouver, Canada). The percentage of LOP will progressively increase over the course of the intervention. Weeks 1 and 2 will be completed at 60% LOP, after which pressure will increase by 5% each subsequent week, corresponding to 65%, 70%, 75%, and 80% LOP during weeks 3, 4, 5, and 6, respectively. The BFR cuffs will remain inflated throughout the exercise protocol, but will deflate for 1 minute every 10 minutes as per safety/tolerability recommendations[54]. The AT-stdCare participants will follow the same training schedule and progression as the AT+BFR group without using BFR cuffs during the exercise sessions. Participants will be eased into the program using a 1-week progressive start; they will complete 63 minutes of exercise in Week 1, split across 3 sessions (21 mins/session). For the remaining 5 weeks, participants will complete 96 minutes of exercise split across 3 sessions per week (32 mins/session). Each exercise session will begin with a five-minute warmup to achieve target intensity and end with a five-minute cool down, neither of which will be counted in the total exercise time. All exercise sessions will be supervised by research staff and take place in a private exercise facility located at each site. To maximize adherence to the intervention, exercise sessions are scheduled on a weekly basis with research staff available 7 days a week.

Data Collection and Management: At the time of first contact with research staff, participants will be assigned a unique identifier (ID), and all files will subsequently be deidentified. Participants will meet with research staff for the purpose of data collection outside of exercise sessions a total of four times: twice at baseline testing and twice at post-testing (Table 1). All data obtained from baseline and post-intervention testing visits will be collected in written form and then transferred to electronic files. All other data collected throughout the intervention will be collected electronically. Physical versions of files will be stored locally in a locked cabinet in a locked room in a restricted access research lab at each site, while digital files will be password-protected and secured on a protected server (REDCap).

Table 1: Measurement Timeline

<table>
<tr><th>Weeks</th><th>1</th><th>2</th><th>Week 3-8</th><th>9</th><th>10</th></tr>
<tr><th>Visits</th><th>1</th><th>2</th><th>3-20</th><th>21</th><th>22</th></tr>
<tr><td>Medical History & Questionnaires</td><td>X</td><td></td><td rowspan="7">6 weeks of aerobic exercise + blood flow restriction (AT+BFR) OR aerobic exercise alone (AT-stCare).

3 visits each week (totalling 63-96 minutes per week)</td><td>X</td><td></td></tr>
<tr><td>Height, Weight, Body Mass Index, Waist-Hip circumferences</td><td>X</td><td></td><td>X</td><td></td></tr>
<tr><td>Body Composition</td><td>X</td><td></td><td>X</td><td></td></tr>
<tr><td>Cardiorespiratory Fitness</td><td></td><td>X</td><td></td><td>X</td></tr>
<tr><td>Dietary Assessment</td><td></td><td>X</td><td></td><td>X</td></tr>
<tr><td>Blood Work</td><td>X</td><td></td><td>X</td><td></td></tr>
<tr><td>Continuous Glucose Monitor</td><td></td><td>X</td><td>X</td><td></td></tr>
</table>

Primary Outcome: The primary outcome of the BOOST-HEALTH Trial is feasibility of a larger definitive trial. Primary selected end-points used to determine feasibility will include: 1) recruitment rates defined as the number of individuals inquiring about the trial, 2) enrollment rates defined as the number of participants who consent to participate in the trial and are randomized to one of the two study arms, 3) adherence to the intervention arms defined as the number of sessions each participant attended during the trial, 4) retention for follow-up testing defined as the number of participants who complete all post-testing measurements after the intervention, and 5) adverse events defined as any undesirable event occurring during the study. We will aim to recruit at least 1 participant per month per site, achieve >70% adherence, retain >80% of participants for follow-up testing, and minimize any adverse events related to BFR or AT.

Secondary Outcomes: *Cardiorespiratory fitness* (VO_{2peak}) will be assessed using a modified Balke and Ware treadmill test protocol. Participants will walk at self-selected speed between 4.5-5.5 kilometers per hour (km/h) at a 0% grade on a motorized treadmill. After 2 minutes, the grade will be increased to 5.0% for 2 minutes, and then progressively increase by 1% every minute until a maximum grade of 15% is achieved. If a participant reaches the maximum grade, then speed will increase by 0.8 km/h each minute until volitational fatigue. Gas exchange will be continuously monitored using a metabolic cart, heart rate data will be obtained throughout the test using a Polar FT1 heart rate monitor (Polar, Kempele, Finland), and blood pressure data will be recorded every 2 minutes using an automated blood pressure monitor. VO_{2peak} will be identified as the average of the highest 30 seconds of VO_2 observed during of the test.

Glycemia will be analyzed through glycated hemoglobin (HbA1c) via Abbott, Afinion™ 2 system analyzer. A finger prick will be conducted using a single-use lancet to collect a 1 microliter

sample of whole blood. The sample will then be analyzed using a point of care analyzer where rapid assessment of HbA1c will be conducted and result provided in approximately 3 minutes. Blood glucose will also be continuously monitored (CGM) using commercially available CGM sensors (Abbott, FreeStyle Libre 3 Plus) for 15 days (7 days before the intervention starts and the first 8 days of the intervention). CGM data will be analyzed using the Diametrics platform[59] yielding outcomes as described in the consensus for reporting of CGM data in trials[60].

Quality of Life will be assessed using validated self-report questionnaires. Health-related quality of life will be measured using the 36-Item Short-Form Health Survey (SF-36) while the diabetes-specific impacts on quality of life will be assessed using select questionnaires from the American Diabetes Association Behavioural Health Toolkit[61].

Exploratory Outcomes: At baseline, research staff will record participant self-reported demographics (age, sex/gender, ethnicity, socioeconomic status, and education), family medical history, T2D duration, and current medication usage. Participants will be monitored throughout the study and asked to report any changes in medication use to research staff as they occur. Medication usage and changes will be confirmed through detailed label inspection (of bottle or photo) or a pharmacy printout.

Physiological and anthropometric measurements will be taken over the span of two days, separated by less than one week. Participants' height, weight, blood pressure, heart rate, and hip and waist circumference will be measured by a member of the research staff according to the Canadian Society for Exercise Physiology protocols[56]. Body composition, including fat mass, lean mass, and body fat percentage, will be estimated using dual-energy x-ray absorptiometry (DXA) following a 12-hour overnight fast.

Blood samples will be collected intravenously from the antecubital vein by a registered nurse or certified phlebotomist into 3mL Vacutainer collection tubes coated with an anticoagulant, ethylenediaminetetraacetic acid (EDTA). The blood samples will then be centrifuged at 1600g for 15 minutes (4°C) and the plasma aliquoted into 1.5mL microcentrifuge tubes for storage at -80°C until further analysis.

Dietary information will be recorded using the Automated Self-Administered 24-Hour Dietary Assessment Tool (ASA24), reporting a weekday and weekend day at both baseline and post-testing to account for potential dietary intake variability[57]. However, participants will be instructed to not change their dietary habits during the study.

Sleep quality will be measured at each time point using the Pittsburgh Sleep Quality Index (PSQI)[58].

Blinding: To maintain single blinding, principal and co-investigators and the statistician will be blinded to participant study groups for data analyses. It is not feasible to blind participants in this trial.

Randomization: Randomization of the intervention participants will occur following completion of the baseline testing visits. Participants will be randomized using a 1:1 allocation ratio with variable permuted block sizes (stratified by sex and site) through the web-based platform REDCap. A member of staff who is not related to the project and has no contact with participants will hold the password-protected randomizations. When a new participant completes baseline testing and is ready for randomization, the participants ID number, sex, and location will be sent to the member of staff with randomizations via email and they will respond to research staff with the randomization (AT+BFR or AT-stdCare.) for that participant.

Sample Size Calculation: There are no clear guidelines for calculating sample size for a pilot/feasibility trial. The sample size calculation was based on determining the feasibility of a larger trial and informed by control group and BFR group data from a study performed by one of our team members using cardiorespiratory fitness as a key efficacy outcome[45]. The mean change in cardiorespiratory fitness for the control and the BFR training group were 1.3% + 4.8% and 8.7% + 7.3%, respectively, from which an effect size Cohen's d was calculated to be 1.2. To be more conservative, using an effect size of 0.25 and nominal type 1 (0.05) and type 2 (0.10) errors, our sample size was estimated to be a total of 46 (n = 23 per arm) for a repeated measure, within-between interaction (calculated using G*Power v3.1). To account for a potential 25% dropout rate, which is typical for exercise trials[62], and missing data, a total sample size of n = 60 (n = 30 per arm; n = 20 per site) was selected.

Informed Consent: Prior to the first baseline testing visit, eligible participants will be provided with a digital copy of the consent form to review. The consent form described in detail the procedures of this study and any benefits and risks associated with their participation. At the beginning of the first testing visit, participants will have time to review a physical copy of the consent form, ask any questions, and consider their participation. If the participant decides to proceed with participating in the study, they will be asked to provide written consent by signing the form, which will be cosigned by research staff. All participants are free to withdraw from the study at any time.

Dissemination: Results from the BOOST-HEALTH trial will be submitted to peer-reviewed journals and presented at scientific meetings. The findings from this study will be used to support and drive future randomized trials exploring the efficacy of BFR training for individuals living with T2D.
